# Exhaustive prediction of disease susceptibility to coding base changes in the human genome

**DOI:** 10.1186/1471-2105-9-S9-S3

**Published:** 2008-08-12

**Authors:** Vinayak Kulkarni, Mounir Errami, Robert Barber, Harold R Garner

**Affiliations:** 1 Mc Dermott Center for Human Growth and Development, UT Southwestern Medical Center, Dallas, TX, USA; 2 Department of Translational Research, UT Southwestern Medical Center, Dallas, TX, USA; 3 Department of Surgery, UT Southwestern Medical Center, Dallas, TX, USA

## Abstract

**Background:**

Single Nucleotide Polymorphisms (SNPs) are the most abundant form of genomic variation and can cause phenotypic differences between individuals, including diseases. Bases are subject to various levels of selection pressure, reflected in their inter-species conservation.

**Results:**

We propose a method that is not dependant on transcription information to score each coding base in the human genome reflecting the disease probability associated with its mutation. Twelve factors likely to be associated with disease alleles were chosen as the input for a support vector machine prediction algorithm. The analysis yielded 83% sensitivity and 84% specificity in segregating disease like alleles as found in the Human Gene Mutation Database from non-disease like alleles as found in the Database of Single Nucleotide Polymorphisms. This algorithm was subsequently applied to each base within all known human genes, exhaustively confirming that interspecies conservation is the strongest factor for disease association. For each gene, the length normalized average disease potential score was calculated. Out of the 30 genes with the highest scores, 21 are directly associated with a disease. In contrast, out of the 30 genes with the lowest scores, only one is associated with a disease as found in published literature. The results strongly suggest that the highest scoring genes are enriched for those that might contribute to disease, if mutated.

**Conclusion:**

This method provides valuable information to researchers to identify sensitive positions in genes that have a high disease probability, enabling them to optimize experimental designs and interpret data emerging from genetic and epidemiological studies.

## Background

The Human Genome Project [1] was driven by the hope that characterization of the human genome would elucidate the molecular etiology of human disease. This resulted into an abundant amount of data regarding the genetic variation across the human genome as amended by the HapMap project [2], which catalogs the location and linkage information for many human genetic variants. The most common variations are single nucleotide polymorphisms (SNPs), single base pair positions in the genome at which different sequence alternatives (alleles) exist. They occur approximately once every 1,000 bases unevenly distributed across the human genome, principally in non-coding regions presumably due to higher selection pressure in coding regions [3]. While approximately half of the SNPs in coding regions are silent [4,5], the other half result in missense mutations (change in the encoded protein sequence) that may be neutral or involved in a disease or phenotype.

SNPs like other genetic variations may be indicators of susceptibility to polygenic diseases [6,7] and could provide a basis for diagnostic and optimal therapeutic choices. A major challenge in realizing these expectations is to identify variants likely to be disease related. While the characterization of all SNPs through disease association studies is economically and practically unrealistic, computational methods to rank SNPs based on their potential impact would help to select and focus on those base positions predicted to be strongly associated with disease. To this end, a variety of approaches with different philosophies have been proposed. Some are purely based on sequence information including conservation in inter-species homologous proteins (orthologs), natural selective pressure at the residue level and the nature of the residue change [8-11]. Other methods combine protein sequence with other physico-chemical properties including protein thermodynamic stability and structure, adding more functionally-relevant knowledge but restricting the applicability of those algorithms to particular cases (known proteins, known structures...) [9,12-16]. An interesting example has been proposed by Fleming *et al*. [17] where the authors categorize missense mutations in the exon 11 of the BRAC1 gene by assigning a probabilistic score representing their likelihood to disrupt the gene function, potentially resulting in breast cancer. The authors used the SIFT (Separating Intolerant from Tolerant) method, considered a gold standard for the prediction of functional effects of mutations [9]. However, this effort has limitations. For instance, SIFT is based on amino acid substitutions and cannot be directly applied to sequences that are transcribed but not translated also called non-coding RNA (ncRNA). These ncRNAs, have been shown to play diverse functions (mRNA splicing, RNA modification, translational regulation etc...) and represent the majority of the transcriptional output in higher eukaryotes [18]. Futhermore, most methods including SIFT, predict effects of non-synonymous substitutions while recent studies have shown that synonymous substitutions, although not resulting in a change in the encoded transcripts may none-the-less cause a measurable phenotypic change and sometimes disease [19].

In this study we propose a predictive method primarily based on sequence and phylogenic information with the aim to apply it to the entire human genome. This method like others hinges on the assertion that evolutionarily conserved nucleotide bases are important for gene function and that single base mutations at these conserved positions are likely to represent disease alleles [20]. Our goal is to predict the impact of a mutation appearing in any gene and at any position, including transcribed but not translated genes, and provide the disease likelihood associated with such a change. Along with the level of interspecies conservation and residue change, we also investigated several factors known or suspected to be disease markers in other published studies such as the location in the protein and nucleotide position in the triplet type of substitution. We have incorporated all these factors into the design of a Support Vector Machine (SVM) classifier that was trained on disease related mutations as found in Human Gene Mutation Database (HGMD) [21] and mutations found in dbSNP [22] for the vast majority of which no disease linkage has been established and are likely to be neutral or of minor phenotypic impact. The SVM classifier appropriately scales these factors relative to their importance and quantifies probable disease-causing substitutions within the human genome regardless of whether they have been previously observed and annotated as such.

## Results and discussion

### Inter-species conservation as a measure of disease susceptibility

**Figure 1:**
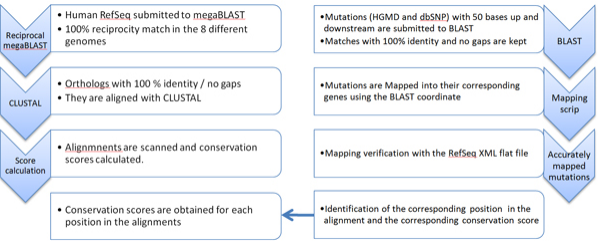
Ortholog identification with reciprocal MegaBLAST, and alignment calculation.

**Figure 2:**
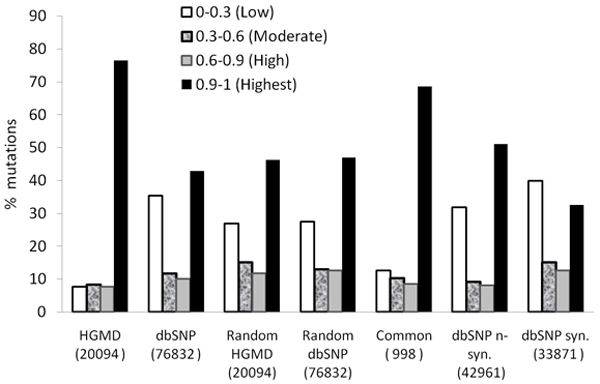
**Distribution of mutations by dataset and conservation score. The bins represent the normalized conservation score levels**. The bins were set in to order to best simplify the figure. The random pools were position selected from that alignments obtained with each pool. For instance for random HGMD: for each HGMD mutation, a position was randomly selected within the same gene (to avoid an alignment bias) leading to a same number of random positions (20094). The common group represents mutations that were found in both datasets dbSNP and HGMD.

Using the process detailed in Figure 1, we have exhaustively confirmed that known disease mutations in HGMD are disproportionately distributed in the most conserved nucleotide positions within genes (Figure 2). The average normalized conservation score calculated for HGMD mutations is 0.88 ± 0.24. Importantly, 87% of HGMD mutations score higher than the gene (average of all positions) in which they occur. Conversely, and as anticipated, dbSNPs mutations tend to occur in the least conserved positions (0.57 ± 0.40, and 0.63 ± 0.40 if synonymous mutations are excluded). Only half of the mutations in dbSNP score higher than the gene in which they occur (57% if synonymous mutations are not considered). The conservation score distribution for randomly selected positions is similar to dbSNPs mutations: the average score is 0.61 ± 0.38 and the fraction of positions scoring higher than the gene is 56%. Since dbSNP and HGMD databases are not cross referenced, it is unknown if the mutations in dbSNP are entirely unique or were co-deposited in both databases. While it seems counter-intuitive that random position have a slightly higher score than dbSNP mutations, the difference is minor compared to what is seen with HGMD mutations or the common set of mutations. The average conservation score calculated for the 998 mutations that were found in both sets (0.80 ± 0.32) confirms the relationship between conservation and sensitivity of a position.

As an interesting aside, we discovered by inspection that the conservation score decreased in the base positions on either side of the nucleotides that were annotated as disease-causing in HMGD. This trend was observed for 83% of HMGD SNPs for up to 5 bases (the maximum number inspected) on either side of the disease-causing base. This suggests that disease-causing alleles have stronger selection pressure relative to the surrounding sequence space.

Finally and as a limitation, under the scoring scheme used, we do not take into account positions in distant species that appear to be conserved, but were in fact mutated multiple times to ultimately revert back to the same nucleotide base. In this particular case, our scoring method doesn't factor in such 'back and forth' mutations.

### Translation information as a measure of disease susceptibility

This study was predominantly aimed at identifying genome-wide factors that could be used to predict disease-like mutations across all genes. Factors associated with mutation position in the codon, residue-change effects, such as alteration of the shape, size or charge of the residue were found to be very weak predictors. However these factors may be of interest in certain subsets of genes. For instance, hydrophobic to hydrophilic substitutions captured in the PHAT matrix [23] constructed specifically to study membrane proteins would presumably be more appropriate to study genes coding for membrane proteins [24].

The BLOSUM and PAM matrices computed from genome-wide alignments of different families of proteins were also found to be useful to segregate sensitive from insensitive base positions. The BLOSUM80 matrix was found to be the most efficient at identifying disease-causing mutations. 94% of HGMD mutations had a non-favorable substitution versus only 45% for dbSNP. This is consistent with the assumption that non-favorable changes may significantly impact the protein function.

### Important predictors and SVM performance

**Figure 3:**
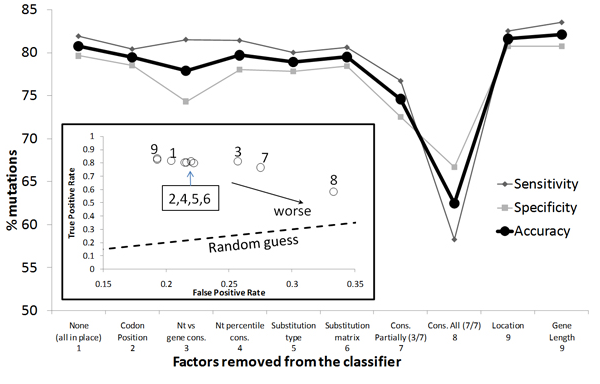
**Leave one factor technique to identify the important factors in the SVM classifier**. Abbreviations include Nt = nucleotide and cons = conservation. Bottom left square: Receiving operating characteristic (ROC) graph. The number next to each point represents the parameter left out from the SVM.

Inter-species conservation information was found to be the strongest predictor among the ones that were selected to identify disease allele. This information was provided to the SVM via 7 different metrics (see Methods). We evaluated the relative importance of these factors using the "leave one factor out technique" (Figure 3). Removal of these factors individually did not significantly affect the classifier performance due to redundant information in the factors. However, removing all of them resulted in a significant decrease in performance. Despite their redundancy, the use of these factors in concert produced the strongest SVM classifier, confirming that inter-species conservation is a crucial parameter for identifying single base mutations that correlate with phenotype alteration.

The other factors found to be good predictors are the BLOSUM 80 substitution matrix, the number of orthologs found for a gene, and the position of the base within the codon (Figure 3). Several random factors were incorporated into the design of the SVM as control parameters (length of the gene, location within the gene). The insignificance of these random factors was confirmed when their removal from the SVM model did not impact its performance.

The final classifier yielded 83% sensitivity and 84% specificity in segregating the HGMD-like mutations from the dbSNP-like mutations. If parameters not related to translation information are removed (codon position, substitution type, substitution matrix) the classifier still achieves 64% sensitivity and 72% specificity. For the majority of misclassified HGMD mutations, we observed that although they had high normalized conservation scores, they were located in genes with few orthologs (less than 3). The classifier is likely to achieve better performance as the number of sequenced genomes and identified orthologs increase. Another set of misclassified HGMD mutations was the 49 synonymous mutations, presumably due to the low number of such mutations in the HGMD training sets. Classification of synonymous mutations will likely improve as more become available in the HGMD.

Unfortunately and although HGMD has now doubled to over 40,782 mutations, it is no longer openly available  limiting us to the last edition publicly available (2005). Finally, when applied to dbSNP, 16% of the SNPs (12,393) were found to have HGMD-like signatures. While these are technically misclassified, they nonetheless remain potential candidates for further investigation. Due to the absence of disease linkage information in dbSNP, we have assumed that most mutations in dbSNPs may not have a significant phenotypic impact. However absence of evidence is not evidence of absence and dbSNP very likely contains a number of disease causing mutations that have as yet to be identified. Identifying them and refining the training sets could also significantly improve the algorithm effectiveness.

### Comparison with SIFT

To assess the performance of the SVM classifier, we compared it with SIFT, a popular method to predict mutations impacts. The last public version of SIFT predictions on dbSNP mutations was downloaded and predictions compared with ours. Out of the 27,279 mutations present in SIFT, only 19,276 were common between the two versions of the database. We also compared the predictions of SIFT for the subset of SNPs present in HGMD, to evaluate the sensitivity and specificity of both methods.

Our SVM, complete and with conservation only were compared to SIFT. Mutations used for testing were different from those used to train our SVMs (Table 1). On these sets, the complete SVM achieves the highest Sensitivity (71% versus 39% for SIFT), at the expense of a lower specificity (62% versus 73%). Overall the best balance of specificity and specificity as measured with the F-measure appears to be achieved when using our SVM, although under these experimental conditions, SIFT achieves the best accuracy (71.9% versus 62.7%). A combination of the SVM-complete and the SIFT method may represent the best alternative. The SVM with conservation only performs at the lowest level.

**Table 1:** SVM performance during testing (labeled with the keyword 'Max') and comparison with SIFT predictions.

	HGMD		dbSNP					
					
	**TP**	**FN**	**TN**	**FP**	**Sensitivity**	**Specificity**	Accuracy	F-measure
	***Testing set created using random mutations not used for training***							

**SVM Complete ; Max**	16527	3567	64378	12454	82.2	83.8	83.5	67.4
**SVM Conservation Only ; Max**	14467	5627	49178	27659	72.0	64.0	65.7	46.5

	***Testing set created to allow comparison with SIFT***							

**SIFT**	159	245	13995	5281	39.4	72.6	71.9	5.4
**SVM Complete**	287	117	12049	7227	71.0	62.5	62.7	7.2
**SVM Conservation Only**	230	174	10216	9060	56.9	53.0	53.1	4.7

Abbreviations: TP = True Positives; FP False Positives; TN = True Negatives; FN = False Negatives.

**Figure 4:**
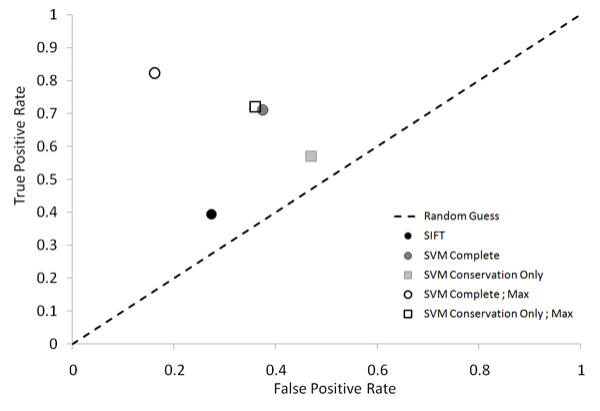
**ROC graph for the comparison of our SVM performance during testing (empty shapes) and during comparison with SIFT (full shapes)**. The points labeled 'Max' represent the performances obtained during testing of our SVM on larger samples of mutations that were not used during training.

Reasons for the diminished performance for our SVM when applied to the specific subset of mutations present in both our data set and that used in the SIFT study is unclear. One contributing factor is the very small overlap in the data for the disease causing mutations, i.e. those found in HGMD, common to both SIFT and our SVM. Since SIFT is computed only on dbSNP mutations, only that small subset of mutations (998 out of 21,946) that appear in both databases can be used for testing and comparison. We specifically excluded these common mutations while training our SVM so that we could best differentiate between and classify mutations that are disease causing (HGMD) and those presumed to be non-disease causing (dbSNP). When compared to the results obtained during the testing of our SVM, the SVM complete achieves an accuracy of 83.5%, with a testing set that contained many more mutations and presumably more illustrative of the actual performance of our SVM (Figure 4).

### Genome wide application and validation

In its present form, the SVM, through the use parameters like BLOSUM80 substitution scores or the base position in the codon, partially depends on translation information limiting its application to genes with well defined expressed sequences (introns/exons, coding frames etc...). Furthermore, the use of BLOSUM80 requires prior knowledge of mutation transitions from their wild type state to their mutated states. Finally, for the 5,073 out of the 39,218 known RefSeq genes that do not encode proteins, it would be inconsistent and unreliable to use codon transitions for predictions.

**Figure 5:**
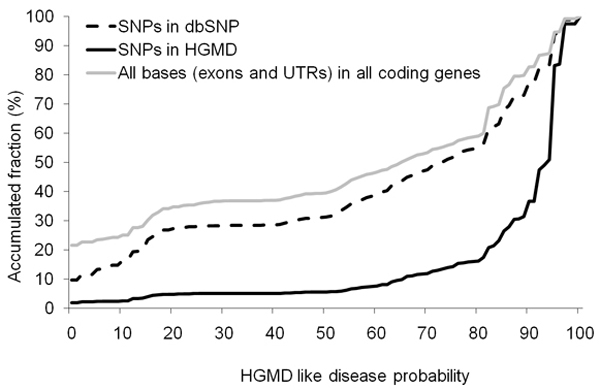
Cumulative frequency distribution as a function of the HGMD-like probability score, with 100% being the most HGMD-like.

Parameters depending on translation information have been removed from a second version of our SVM that has been applied to all the coding regions within the human genome. Each base position has been scored in accordance to the disease likelihood associated with a mutation occurring at this particular position. The calculated score provides a quantitative measure scaled from 0 to 100 instead of a qualitative prediction (disease/non-disease). Figure 5 shows the cumulative frequency distribution for all HGMD and dbSNP mutations as a function of HGMD like score (with 100% being the more HGMD like). The distribution for all coding bases in the human genome is also shown for comparison. The similarity between the dbSNP accumulated distribution and all coding bases, suggest that most bases, if altered, will not result in disease causation (i.e. are not HGMD-like). The HGMD distribution curve was used to identify a threshold corresponding to the inflection point with maximum slope (maxima of the derivative). This threshold was found to be 94%. This 6% range of conservation (from 94% to 100%) is enriched with over half of the mutations in HGMD, while it includes only 13% of nucleotide bases (or 12,038,565 bases) in the entire coding genome that could potentially play a causative role in disease/phenotype alteration. Mutations and SNPs occurring within this range of conservation score, and for which no disease linkage has been established, have the highest potential value for researchers designing disease association studies.

Using our methodology 26,874 gene transcripts in the human genome found at least one ortholog in comparable genomes. These genes were then ranked according to their likelihood of being associated with disease if mutated, based on our disease probability score obtained with our SVM based on conservation information only. Out of the 30 top scoring genes, which are very sensitive to variation, 21 are known to be directly associated with a disease from published literature (Additional file 1). Conversely, only one gene out of the 30 with the lowest scores were found to be associated with disease in the published literature. Many of those genes with lower scores are hypothetical, likely due to the low similarity with known genes (consistently with low conservation and low scores). The results strongly suggest that the highest scoring genes are indeed enriched with those that might contribute to disease if mutated.

## Conclusion

We have developed a comparative genomic analysis method for genome-wide identification of genome positions with a greater likelihood of being important to gene function. Mutations occurring at these sites have a higher probability of representing disease alleles. New single base mutations or SNPs can then be scored for their potential to cause a disease, helping direct SNP discovery efforts.

There is an underlying signature for disease mutations, as evidenced by their occurrence in the most conserved nucleotide positions, the favorability of residue substitution observed from BLOSUM scores, codon bias and other factors. This signature was exploited to identify and flag putative disease-causing mutations in all coding human bases, including some in dbSNP, irrespective of the existence of annotation as causing a disease or clinical phenotype. Additional genetic (or other) factors, if discovered in the future to be causative of disease, can be easily incorporated in the prediction algorithm, i.e. the SVM classifier. This makes this tool and approach versatile, enabling one to quantitatively test the strength of new factors or metrics for their potential for disease causation.

## Methods

### Overview and basic data sets

Our predictive method is based on a Support Vector Machine (SVM) algorithm that one can easily implement using the popular 'R' statistical software tool  and more importantly that satisfactorily addresses the non parametric nature of the data distribution for a number of individual input factors [25]. Those factors, including different inter-species conservation parameters, were chosen for their ability to identify positions in the genome where mutations may have a significant phenotypic impact (i.e. disease). Figure 1 represents the process established to calculate the conservation score for every nucleotide in the human genome.

Genes were obtained from the Reference Sequence (RefSeq) Database (Release 21) [26]. Single base mutations were obtained from Human Gene Mutation Database (HGMD)[27], forming a set of disease causing mutations (last available public version, June 2005, 21,964 mutations) and from dbSNP [22] (build 126, 97,102 mutations in the coding regions) that is assumed to contain for the vast majority neutral mutations. The mutations were extracted from ENTREZ SNPs page  setting the limits parameters to {Organism : Homo Sapiens, Functional class : coding non synonymous, coding synonymous, mRNA UTR and only reference SNPs, SNP class : SNP}. A subset of 998 redundant mutations between both was separated into the 'Common SNPs' set and later used as one of the tests of the SVM classifier. Mutations in dbSNP were partitioned into synonymous (33,871 mutations) and non-synonymous (42,961 mutations).

### Inter-species alignment and conservation score

#### Finding putative human gene orthologs in other species for optimal alignment

For every human RefSeq entry, its corresponding ortholog was identified using megaBLAST [28] in a reciprocal manner . Briefly, each human RefSeq was BLASTed against eight comparable vertebrate genomes Table 2, and the highest scoring similar sequence for each genome was reverse-BLASTed against the human RefSeq database. Only those non-human sequences for which the original human sequence was the highest scoring match were retained as putative orthologs (Table 2). Each human RefSeq was subsequently aligned with its putative orthologs using CLUSTALW [29].

**Table 2:** Human RefSeq orthologs found in other species using reciprocal MegaBLAST. The number of orthologs found are roughly inversely proportional to the phylogenetic distances (from Human), imported from the UCSC browser.

**Organism**	**Total number of Reference Sequences**	**Putative Orthologs found in Human**	**Phylogenetic distance from Humans**
*Homo sapiens*	39,218	-	0
*Pan troglodytes*	57,924	22,743	1.4
*Macaca mulatta*	43,198	19,907	6.4
*Canis familaris*	33,644	15,783	33.5
*Bos taurus*	26,501	15,519	34.2
*Mus musculus*	50,569	14,665	45.3
*Rattus norvegicus*	40,672	14,168	46.1
*Gallus gallus*	19,131	5,191	108.7
*Danio rerio*	35,695	1,789	182.9

#### Evaluating nucleotide conservation

**Figure 6:**
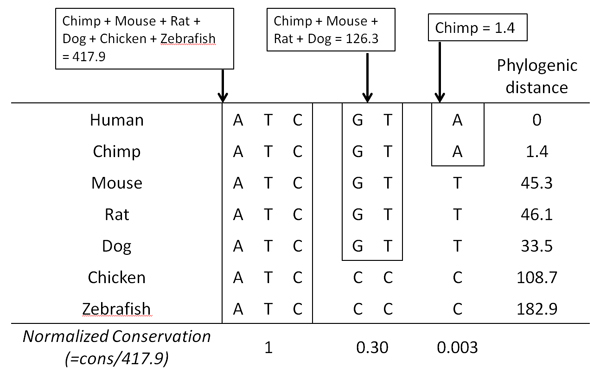
Conservation score calculated as the weighted average of phylogenic distances in conserved genomes.

Ideally, a conservation scoring method would result in significantly higher scores at 'sensitive' positions where disease causing mutations occur comparatively to random positions. For each position in each gene, a score was calculated as the weighted average of the conservation obtained from the alignment of this gene and its orthologs. The weighting parameters were the UCSC browser phylogenetic distances [30] for the genomes where the given nucleotide is conserved and were kindly provided by the UCSC genome browser group (Table 2). In this way, a nucleotide conserved in a distant genome weighs more than the same nucleotide conserved only in closely related species. This score has also been normalized to allow comparisons between genes (for each gene, the number of orthologs will be different) (Figure 6).

To ensure that conservation scores significantly correlate with disease alleles, normalized conservation scores were also computed for randomly selected base positions in genes containing mutations listed in HGMD or dbSNP databases (1,260 and 14,449 genes, respectively, Table 3). As shown in Figure 2, disease-causing mutations occur at highly conserved positions and usually are the most conserved positions within the gene. Conversely, and as anticipated, dbSNPs mutations tend to occur in the least conserved positions.

**Table 3:** Total mutations mapped and genes represented in both datasets.

	**HGMD**	**dbSNP**
Total coding mutations in the database	21,964	97,102
Total mutations correctly mapped	20,094	76,832
Genes represented	1,260	14,449

### Mapping SNPs from HGMD and dbSNP to their gene location

Each and every mutation in the two datasets HGMD and dbSNP, were mapped to their gene location in three steps. 1) For each mutation, 50 base flanking regions upstream and downstream were BLASTed against the human genome. Only hits with 100% identity and no gaps were kept. 2) The BLAST coordinates were used to map the exact location of the given mutation nucleotide within the gene. 3) In order to ensure accurate mapping of mutation positions to the genes, mutations obtained from dbSNP were cross-validated using the Genbank XML flat file and mutations obtained from HGMD were cross-validated using upstream and downstream flanking regions. Using this procedure, a total of 20,094 and 76,832 unique mutations from HGMD and dbSNP, respectively, were accurately mapped, as shown in Table 3.

### SVM design

A number of factors that we hypothesized may quantify the effect of a mutation, including conservation, location of the mutation within the gene, substitution type (synonymous or non-synonymous), type of residue change in terms of charge, mass, volume, hydrophobicity, and favorability of a substitution as represented in the BLOSUM and PAM matrices were used in the SVM. For these SVMs, the radial kernel function was used to design the classifier due to the non-linear nature of the parameters with cost and gamma parameters set as 1 and 0.125 respectively to enhance the classifier performance.

#### Conservation

**Figure 7:**
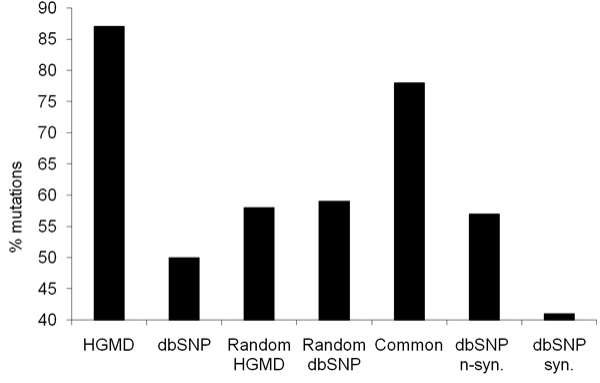
Proportion of mutations with a conservation score higher than the average score of the gene in which they occur.

The conservation information used in the SVM is based on seven different metrics: (1) nucleotide raw conservation score, (2) normalized conservation score to the maximum possible score per position, (3) maximum conservation score per gene, (4) average conservation score per gene (5) percentage of bases with conservation scores below or (6) equal to the nucleotide position of interest, and (7) comparison of nucleotide conservation score with the average conservation score within the gene. Figure 7 shows that in contrast to dbSNP mutations or random positions, HGMD mutations tend to occur at positions that are significantly more conserved than the gene itself (as measured by the conservation averaged over all its positions). Although these different conservation metrics have redundancy, analysis using the 'leave one out' technique will identify which of these metrics provide the best ability to separate disease causing base variants (from HGMD) from those base positions unlikely to be associated with disease (from dbSNP).

#### Parameters relying on availability of translation information

Although the method we propose is not dependent upon translation information, various parameters including amino-acid substitution matrixes were considered in order to assess their relative importance in predicting mutations impact at various positions. Furthermore, the use of translation information (when available) facilitates the comparison of at least a portion our method with SIFT.

Two sets of widely used matrixes, BLOSUM [31] and PAM [32] matrices (BLOSUM30, BLOSUM60, BLOSUM62, BLOSUM80, BLOSUM90, BLOSUM100 PAM10, PAM50, PAM100, PAM250, and PAM500) were tested for every mutation in both dbSNP and HGMD datasets to determine whether distant or close relationship-based matrixes are more appropriate for identification of base positions likely to cause a disease if mutated. Again, it is reasonable to expect that disease-causing mutations found in HGMD would more often result in unfavorable substitutions than mutations in dbSNP.

A number of physico-chemical matrixes have been used to take into account possible aberrant changes in the volume, mass or charge of the original amino acid that could potentially disrupt the protein structure. Any extreme change in any of the above factors could have a disproportionately high impact and therefore be overrepresented in disease causing alleles (i.e., mutations in HGMD relative to mutations in dbSNP).

Each individual mutation was also annotated for its location within the codon. Mutations at the first position are more likely to modify the protein sequence than mutations occurring at the second and third positions. Consequently mutations at the first codon position were expected to occur more often in HGMD than in dbSNP.

#### SVM training and testing

**Figure 8:**
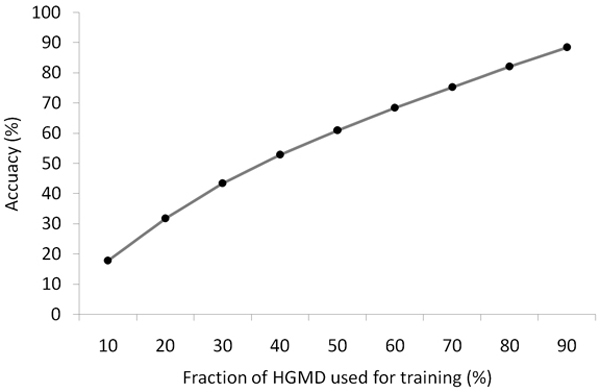
SVM accuracy as a function of the training set size.

All the above-mentioned factors were individually evaluated for their potential to identify diseased alleles and statistically segregate HGMD from dbSNP mutations. The set of 19,096 HGMD mutations (20,094 minus the 998 redundant) was randomly split in two subsets for training (80% or 15,277 mutations) and testing (20% or 3,819 mutations). An equal number of mutations from dbSNP were also randomly chosen and mixed with the corresponding HGMD subset. This 80–20 proportion has been chosen as a good comprise between the size of the testing and training sets, and to allow us to obtain a testing set big enough for comparison with SIFT. The obtained accuracy with these proportions is 84.3% (Figure 8).

The importance of each factor was weighed individually against the final prediction outcome by employing the 'leave one factor out' method (Figure 3). Only factors that significantly contributed to the prediction outcome were kept to establish a final SVM classifier.

#### SVM validation

The final classifier was applied to all coding bases within the human genome to identify high impact positions, where mutations might have a higher probability for disease-causation. Literature, ontologies and pathway relationships for genes with highest and lowest base-averaged SVM scores were further inspected. GeneSifter (VizX Labs, Seattle, WA), a software program typically applied to the analysis and interpretation of gene expression data, was used to generate GO Ontology information. Pathways analysis reports were obtained from the Kyoto Encyclopedia of Genes and Genomes (KEGG) [33]. Disease associations were also based on annotations provided by the NCBI, the Stanford SOURCE database [34], the Comparative Toxicogenomic database [35] and published literature. Similar analyses were performed with genes randomly selected as a control, thus allowing the calculation of a z-score.

## Competing interests

The authors declare that they have no competing interests.

## Authors' contributions

Vinayak Kulkarni developed the pipeline for SNP analysis, including prepping all the data from different datasets, annotating the SNPs with additional information from various sources and designing and fine-tuning the classifier for optimal prediction. Mounir Errami analyzed the data, lead the project and the preparation of this article. Robert Barber provided with valuable inputs at various time points of the project which helped in validating the hypothesis and build a robust method. Harold Garner provided guidance and organizational support for the successful completion of the project.

## Supplementary Material

Additional file 1 Supplementary Table: Genes with the highest and lowest HGMD like scores involved in a disease, as per published literature. The score is the disease association probability (the maximum being 100). Click here for file
