# Supplementary material for: Exhaustive prediction of disease susceptibility to coding base changes in the human genome
Source: BMC Bioinformatics. 2008 Aug 12;9(Suppl 9):S3. doi: 10.1186/1471-2105-9-S9-S3 (PMC2537574; doi:10.1186/1471-2105-9-S9-S3)
Supplement: Additional file 1 — Supplementary Table: Genes with the highest and lowest HGMD like scores involved in a disease, as per published literature. The score is the disease association probability (the maximum being 100). [file 1471-2105-9-S9-S3-S1.doc]

**Supplementary Table: Genes with the highest and lowest HGMD like scores involved in a disease, as per published literature. The score is the disease association probability (the maximum being 100).**

| **Refseq ID** | **Score** | **Gene Name** | **Disease** | **Ref(s)** |
| --- | --- | --- | --- | --- |
| ***30 Genes with highest scores*** | | | | |
| NM_005826 | 91.39 | (HNRPR) | Spinal muscular atrophy | [1] |
| NM_004189 | 91.20 | SRY (sex determining region Y)-box 14 (SOX14) | Cancer | [2;3] |
| NM_005444 | 90.89 | Required for cell differentiation1 (RQCD1) | Non Hodgkin lymphoma | [4] |
| NM_004465 | 90.58 | Fibroblast growth factor 10 (FGF10) | ectodermal dysplasia | [5;6] |
| NM_003868 | 90.18 | Fibroblast growth factor 16 (FGF16) | ectodermal dysplasia | [7] |
| NM_020660 | 90.09 | Connexin-36 (CX36) | amyotrophic lateral sclerosis (ALS) | [8] |
| NM_000829 | 89.88 | Glutamate receptor, ionotrophic, AMPA 4 (GRIA4) | Ewing sarcoma | [9] |
| NM_000217 | 89.80 | Potassium voltage-gated channel (KCNA1) | myokymia with periodic ataxia | [10] |
| NM_015384 | 89.79 | Nipped-B homolog (NIPBL), transcript variant B | Limb defects | [11;12] |
| NM_012433 | 89.72 | Splicing factor 3b, subunit 1, 155kDa (SF3B1) |  |  |
| NM_005294 | 89.60 | G protein-coupled receptor 21 (GPR21) |  |  |
| NM_001013732 | 89.58 | Chromosome 6 orf 138 (C6orf138) |  |  |
| NM_024409 | 89.45 | Natriuretic peptide precursor C (NPPC) | Congestive Heart Failure / Kidney Failure | [13] |
| NM_003688 | 89.40 | Calcium/calmodulin-dep serine kinase(CASK) | Cornelia de Lange syndrome | [14] |
| NM_005288 | 89.36 | G protein-coupled receptor 12 (GPR12) | Dwarfism and early death (mice) | [15] |
| NM_001358 | 89.35 | DEAH box polypep. 15 (DHX15) | Prostate cancer | [16] |
| NM_004714 | 89.35 | Dual-specificity YP regulated K 1B (DYRK1B) | Juvenile myoclonic epilepsy, Deafness | [17] |
| XM_001133072 | 89.26 | Ubiquitine Conjugation Enzyme E2E (UBE2E3) | Prostate cancer | [18] |
| NM_178862 | 89.18 | Oligosaccharyltransferase (STT3B) |  |  |
| NM_015037 | 89.17 | KIAA0913 (unknown) |  |  |
| NM_024045 | 89.17 | DEAD box polypetide 50 (DDX50) |  |  |
| NM_032580 | 89.13 | Hairy and enhancer of split 7 (HES7) |  |  |
| NM_015028 | 89.11 | Traf2/NCK interacting kinase (TNIK) |  |  |
| NM_005406 | 89.10 | Rho-associated protein (ROCK1) | Cancer | [19] |
| NM_005243 | 89.05 | Ewing sarcoma breakpoint region 1 (EWSR1) | Alzheimer's disease | [20] |
| NM_020449 | 88.98 | THO complex 2 (THOC2) | Epilespy, Deafness | [21] |
| XM_294370 | 88.97 | Guanine nucleotide binding protein (GNAT3) | Achromatopsia | [21] |
| NM_194247 | 88.90 | Ribonucleoprotein A3 (HNRPA3) |  | [22;23] |
| NM_004236 | 88.89 | COP9 homolog subunit 2 (COPS2) | Adrenal hypoplasia congenita | [24] |
| NM_001260 | 88.85 | Cyclin-dependent kinase 8 (CDK8) | Mental retardation | [25] |
| ***30 Genes with lowest scores*** | | | | |
| XM_932558 | 19.36 | Hypothetical LOC644982 (LOC644982) |  |  |
| XM_001134268 | 19.30 | Hypothetical |  |  |
| XM_371461 | 19.30 | Hypothetical KIAA1671 protein (CTA-221G9.4) |  |  |
| XM_001130249 | 19.14 | PRAME family member 19 (PRAMEF19) |  |  |
| NM_001004355 | 19.12 | FLJ31132 |  |  |
| NM_001004305 | 19.06 | Hypothetical protein LOC284757 (LOC284757 ) |  |  |
| XR_017915 | 19.06 | Hypothetical miscRNA (LOC401242) |  |  |
| XM_001130664 | 19.05 | hypothetical protein LOC731756 |  |  |
| NM_007335 | 18.94 | Deleted in lung and esophageal cancer 1 (DLEC1) | Neoplasms | [26] |
| XM_001130862 | 18.93 | FLJ43860 protein |  |  |
| XM_942756 | 18.92 | Hypothetical LOC442124 ( LOC442124) |  |  |
| NM_207432 | 18.89 | Chromosome 11 orf 39 (C11orf39) |  |  |
| XM_001133556 | 18.66 | Hypothetical protein LOC729135 (LOC729135 ) |  |  |
| XR_016958 | 18.42 | Hypothetical LOC645479 (LOC645479) |  |  |
| XM_001130543 | 18.22 | Hypothetical golgi autoantigen |  |  |
| NM_001010913 | 17.75 | Hypothetical |  |  |
| NM_001039776 | 17.05 | Hypothetical protein MGC10701 |  |  |
| NM_020779 | 16.52 | WD repeat domain 35 (WDR35) |  |  |
| NM_003417 | 16.45 | Zinc finger protein 264 (ZNF264) |  |  |
| XM_943032 | 16.44 | Hypothetical LOC647805 (LOC647805) |  |  |
| NM_207367 | 16.05 | Hypothetical LOC346547 ( LOC346547) |  |  |
| NM_175908 | 15.91 | Hypothetical LOC346547 (LOC346547) |  |  |
| XM_001131625 | 15.91 | Hypothetical protein LOC732021 (LOC732021) |  |  |
| XM_001129640 | 15.75 | Hypothetical DKFZp434I1020 (B-dynamin) |  |  |
| NR_002801 | 15.58 | testis-specific transcript, Y-linked 15 (TTTY15) |  |  |
| NR_001545 | 15.51 | Homo sapiens testis-specific transcript |  |  |
| XM_379273 | 15.30 | Hypothetical AL137733 (FLJ14186) |  |  |
| NM_205857 | 15.26 | Chromosome 4 orf 12 (C4orf12) |  |  |
| NM_194439 | 13.83 | Ring finger protein 212 (RNF212) |  |  |
| NM_001013678 | 13.68 | Hypothetical LOC400968 (LOC400968) |  |  |

**References**

1. Hassfeld W et al: **Molecular definition of heterogeneous nuclear ribonucleoprotein R (hnRNP R) using autoimmune antibody: immunological relationship with hnRNP P**. *Nucleic Acids Res* 1998. **26**:439-45.

2. Arsic N, Rajic T, Stanojcic S, Goodfellow PN, Stevanovic M: **Characterisation and mapping of the human SOX14 gene**. *Cytogenet Cell Genet* 1998. **83**:139-46.

3. Cremazy F, Soullier S, Berta P, Jay P: **Further complexity of the human SOX gene family revealed by the combined use of highly degenerate primers and nested PCR**. *FEBS Lett* 1998. **438**:311-4.

4. Okazaki N et al: **Novel factor highly conserved among eukaryotes controls sexual development in fission yeast**. *Mol Cell Biol* 1998. **18**:887-95.

5. Emoto H et al: **Structure and expression of human fibroblast growth factor-10**. *J Biol Chem* 1997. **272**:23191-4.

6. Igarashi M, Finch PW, Aaronson SA: **Characterization of recombinant human fibroblast growth factor (FGF)-10 reveals functional similarities with keratinocyte growth factor (FGF-7)**. *J Biol Chem* 1998. **273**:13230-5.

7. Miyake A et al: **Structure and expression of a novel member, FGF-16, on the fibroblast growth factor family**. *Biochem Biophys Res Commun* 1998. **243**:148-52.

8. Belluardo N, Trovato-Salinaro A, Mudo G, Hurd YL, Condorelli DF: **Structure, chromosomal localization, and brain expression of human Cx36 gene**. *J Neurosci Res* 1999. **57**:740-52.

9. McNamara JO et al: **Chromosomal localization of human glutamate receptor genes**. *J Neurosci* 1992. **12**:2555-62.

10. Freeman SN, Conley EC, Brennand JC, Russell NJ, Brammar WJ: **Cloning and characterization of a cDNA encoding a human brain potassium channel**. *Biochem Soc Trans* 1990. **18**:891-2.

11. Tonkin ET, Wang T, Lisgo S, Bamshad MJ, Strachan T: **NIPBL, encoding a homolog of fungal Scc2-type sister chromatid cohesion proteins and fly Nipped-B, is mutated in Cornelia de Lange syndrome**. *Nat Genet* 2004. **36**:636-41.

12. Krantz ID et al: **Cornelia de Lange syndrome is caused by mutations in NIPBL, the human homolog of Drosophila melanogaster Nipped-B**. *Nat Genet* 2004. **36**:631-5.

13. Tawaragi Y et al: **Gene and precursor structures of human C-type natriuretic peptide**. *Biochem Biophys Res Commun* 1991. **175**:645-51.

14. Hata Y, Butz S, Sudhof TC: **CASK: a novel dlg/PSD95 homolog with an N-terminal calmodulin-dependent protein kinase domain identified by interaction with neurexins**. *J Neurosci* 1996. **16**:2488-94.

15. Saeki Y et al: **Molecular cloning of a novel putative G protein-coupled receptor (GPCR21) which is expressed predominantly in mouse central nervous system**. *FEBS Lett* 1993. **336**:317-22.

16. Ono Y, Ohno M, Shimura Y: **Identification of a putative RNA helicase (HRH1), a human homolog of yeast Prp22**. *Mol Cell Biol* 1994. **14**:7611-20.

17. Leder S et al: **Cloning and characterization of DYRK1B, a novel member of the DYRK family of protein kinases**. *Biochem Biophys Res Commun* 1999. **254**:474-9.

18. Desterro JM, Thomson J, Hay RT: **Ubch9 conjugates SUMO but not ubiquitin**. *FEBS Lett* 1997. **417**:297-300.

19. Inagaki M et al: **Phosphorylation sites linked to glial filament disassembly in vitro locate in a non-alpha-helical head domain**. *J Biol Chem* 1990. **265**:4722-9.

20. Delattre O et al: **Gene fusion with an ETS DNA-binding domain caused by chromosome translocation in human tumours**. *Nature* 1992. **359**:162-5.

21. Scherer SW et al: **Human chromosome 7: DNA sequence and biology**. *Science* 2003. **300**:767-72.

22. Lairmore TC et al: **A 1.5-megabase yeast artificial chromosome contig from human chromosome 10q11.2 connecting three genetic loci (RET, D10S94, and D10S102) closely linked to the MEN2A locus**. *Proc Natl Acad Sci U S A* 1993. **90**:492-6.

23. Takiguchi S et al: **Identification and characterization of a cDNA, which is highly homologous to the ribonucleoprotein gene, from a locus (D10S102) closely linked to MEN2 (multiple endocrine neoplasia type 2)**. *Cytogenet Cell Genet* 1993. **64**:128-30.

24. Lee JW, Choi HS, Gyuris J, Brent R, Moore DD: **Two classes of proteins dependent on either the presence or absence of thyroid hormone for interaction with the thyroid hormone receptor**. *Mol Endocrinol* 1995. **9**:243-54.

25. Schultz SJ, Nigg EA: **Identification of 21 novel human protein kinases, including 3 members of a family related to the cell cycle regulator nimA of Aspergillus nidulans**. *Cell Growth Differ* 1993. **4**:821-30.

26. Daigo Y et al: **Molecular cloning of a candidate tumor suppressor gene, DLC1, from chromosome 3p21.3**. *Cancer Res* 1999. **59**:1966-72.
